# Image-Guided Radiofrequency Hyperthermia (RFH)-Enhanced Direct Chemotherapy of Hepatic Tumors: The Underlying Biomolecular Mechanisms

**DOI:** 10.3389/fonc.2020.610543

**Published:** 2021-01-28

**Authors:** Kun Qian, Minjiang Chen, Feng Zhang, Jeffrey Forris Beecham Chick, Hongxiu Ji, Chuansheng Zheng, Xiaoming Yang

**Affiliations:** ^1^ Image-Guided Bio-Molecular Interventions Research & Division of Interventional Radiology, Department of Radiology, University of Washington School of Medicine, Seattle, WA, United States; ^2^ Department of Radiology, Union Hospital, Tongji Medical College, Huazhong University of Science and Technology, Wuhan, China; ^3^ Hubei Province Key Laboratory of Molecular Imaging of Tongji Medical College, Huazhong University of Science and Technology, Wuhan, China

**Keywords:** radiofrequency hyperthermia, hepatic tumors, heat shock protein 70, chemotherapy, doxorubicin

## Abstract

**Purpose:**

To evaluate the treatment effect of radiofrequency-induced hyperthermia (RFH) combined with intra-tumoral chemotherapy for rabbit VX2 liver tumors and explore the underlying mechanism that drives local hyperthermia-enhanced chemotherapy.

**Materials and Methods:**

VX2 cell lines and rabbits with liver VX2 tumors were randomly allocated to four treatment groups including: (1) combination therapy of Doxorubicin (DOX) plus hyperthermia/RFH (n=6); (2) DOX only; (3) hyperthermia/RFH only (n=6); and (4) phosphate-buffered saline-treated control (n=6). Cell viability and doxorubicin uptake by VX2 tumor cells were assayed using flow cytometry and fluorescence microscopy 24 h after treatments. Western blot was used to evaluate the expression level of heat shock protein 70 (HSP70) in tumor cells and tissues. For the harvested VX2 tumors, fluorescence microscopy was used to evaluate the distribution and penetration of doxorubicin in tumor tissues and HSP70 expression was analyzed by Western blot and immunohistochemistry.

**Results:**

RFH enhanced the chemotherapeutic effect of doxorubicin in VX2 cells and rabbit liver VX2 tumors resulting in higher apoptosis and lower cell viability. Flowcytometry of VX2 cells showed more apoptotic cells in combination therapy of hyperthermia and DOX, compared with other three groups in-vitro experiments (45.80 ± 1.27% vs 20.66 ± 0.71%, vs 15.16 ± 0.81% and 0.62 ± 0.06%, respectively, p<0.01). The quantitative analysis by Western blot and immunohistochemistry showed increased expression of HSP70 in both VX2 tumor cells (1.28 ± 0.13 vs 0.64 ± 0.13 vs 0.83 ± 0.10 vs 0.15 ± 0.03, respectively, p<0.05) and tumors (1.47 ± 0.13 vs 0.51 ± 0.13 vs 0.74 ± 0.11 vs 0.16 ± 0.04, respectively, p <0.01). Fluorescence microscopy showed increased uptake of DOX in tumor cells in the combination therapy group.

**Conclusions:**

RFH/hyperthermia enhanced the chemotherapeutic effect of DOX in VX2 tumors by promoting the uptake of DOX and the expression HSP70 in tumors.

## Introduction

Hepatocellular carcinoma (HCC) is a major cause of cancer-related death in the world (1). Despite improvements in surveillance programs and diagnostic techniques, most patients are diagnosed at the intermediate-to-advanced stages and only a small percentage of newly diagnosed patients are eligible for curative treatments such as ablation, surgical resection, and liver transplantation.

Imaging-guided radiofrequency ablation (RFA) is currently used as an alternative to surgery in well-selected patients and is considered a safe and effective treatment for patients with early-to-intermediate HCC (3–5 cm) when surgical resection is not feasible (2). As tumor size increases; however, there are high local recurrence and tumor progression rates, particularly for tumors beyond 5-cm. Residual tumor cells, particularly at the margins, that may not be completely ablated, may be sources of local tumor progression due to sublethal thermal injury (3).

For RFA of tumors larger than 5 cm, uniform necrosis throughout the entire tumor is unlikely and due to a lower thermal dose in the margins of tumors. Radiofrequency (RF) ablation induces ablative temperatures >70°C in the tumor around the RF probe, but with the increasing distance from the tip, there may be thermal energy attenuation.

Chemotherapeutic effects on tumors may be significantly enhanced by radiofrequency-induced hyperthermia (RFH) (4, 5). Previous studies showed that at the margin of an RF-ablated liver tumor, the measured temperatures were 42–44°C. This sublethal hyperthermia may enhance the therapeutic effects of chemotherapeutic drugs infused through RF needles 6–8 with the goal of completely eliminating the viable tumor cell in the marginal regions.

This goal of this study was to evaluate the treatment effect of RFH combined with intra-tumoral chemotherapy for rabbit VX2 liver tumors and explore the underlying mechanism that drives local hyperthermia-enhanced chemotherapy.

## Materials and Methods

### Cell Culture

VX2 cells, purchased from cell resource center for biomedical research (IDAC, Tohoku University, Japan), were seeded in 6-well plates (1×105 per well) (Thermo Fisher Scientific; Rochester, NH). Cells were maintained in RPMI-1640 medium (HyClone Laboratories; Logan, UT) supplemented with 10% fetal bovine serum (HyClone Laboratories) at 37°C in 5% CO2 atmosphere.

### Hyperthermia-Enhanced Chemotherapy on VX2 Cells and *in Vitro* Experiments

The cells were treated with (1) combination therapy of doxorubicin (DOX, 5μM) with hyperthermia at 42 ± 1°C for 20 min; (2) DOX alone; (3) hyperthermia alone; and (4) phosphate-buffered saline (PBS) to serve as the control. The dose of DOX (Mylan Institutional LLC; Rockford, IL) for cell treatment was determined by the 50% inhibitory concentration (IC50).

**Figure 1:**
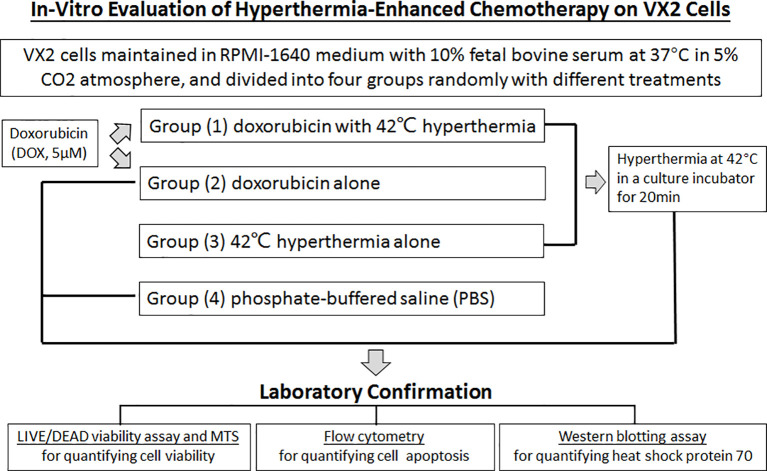
The flow of in-vitro confirmation on hyperthermia-enhanced chemotherapy of VX2 tumor cells.

The VX2 cell lines were cultured in complete media with or without Doxorubicin (5μM) in mid-plastic Petri dish and heat stressed at the temperatures of 42°C± 1°C in a culture incubator (Type 37900 culture incubator, Thermolyne, USA) (**Figure 1**).

### Cell Viability and DOX Uptake Assay

Cell viability was evaluated by subjecting treated cells to LIVE/DEAD Viability Assay using a Cell Viability Imaging Kit (Thermo Fisher Scientific). The viability and DOX uptake of VX2 cells were evaluated by fluorescence microscopy (IX73, Olympus America) which showed a green fluorescence in live cells and a red fluorescence in dead cells. The blue fluorescence of DAPI (4’,6-diamidino-2-phenylindole staining of the cells nuclear DNA) and red fluorescence signal intensity of DOX were measured in six randomly selected fields per well (% of total area). Quantitative analysis was evaluated by MTS [3-(4,5-dimethylthiazol-2-yl)-5-(3-carboxy-methoxyphenyl)-2-(4-sulfophenyl)-2H-tetrazoliu] assay (Promega Corporation; Madison, WI), and measured at 490-nm with a microplate reader (VersaMax; Molecular Devices, Sunnyvale, CA). Relative cell viability of the four groups was calculated using the equation of A_treated_−A_blank_/A_control_−A_blank_; where A is the absorbance of formazan.

### Apoptosis Analysis by Flow Cytometry

Cell apoptosis was quantified by staining the cells with annexin V/propidium iodide (PI) Dual Staining Kit (BD Biosciences; San Diego, CA). Twenty-four hours after treatments both adherent and floating cells in the four groups were incubated with a mixture of annexin V reagent and propidium iodide. Samples were analyzed by a FACScan flow cytometer (BD Biosciences; San Jose, CA). Data were analyzed using the FlowJo software version 10 (FloJo Data Analysis Software; Ashland, OR).

### Western Blotting Assay for Quantifying Heat Shock Protein 70 (HSP70) in Cells

Twenty-four hours after the treatment, cells in each group were collected for protein extraction. The concentration of protein was measured with the Pierce BCA Protein Assay Kit (Thermo Fisher Scientific). The proteins were separated by gel electrophoresis using a 4–12% standard electrophoresis sodium dodecyl sulfate-polyacrylamide (SDS-PAGE) gel and then transferred onto polyvinylidene difluoride (PVDF) membranes (Thermo Fisher Scientific). Membranes were blocked in 5% defatted milk for 2 h at room temperature and incubated overnight at 4°C with the following antibodies: anti-Hsp70 (1:1,000, ab2787, Abcam; Cambridge, MA) and anti-GAPDH (1:1,000, Sigma) for 2 h at room temperature followed by incubation with a secondary antibody (1:5,000, Sigma). The level of protein expression was quantified by the intensity of the bands corresponding to the immunoreactive proteins using Quantity One software (Bio-Rad)s and normalized using Glyceraldehyde-3-Phosphate Dehydrogenase (GAPDH) for comparison.

### *In Vivo* Experiments

The Institutional Animal Care and Use Committee approved these animal experiments. Twenty-four female New Zealand White rabbits (2.5–3.5 kg) were used for the *in vivo* experiments. Rabbits were anesthetized with inhalation of isoflurane delivered in oxygen at the flow of 1 l/min.

### Creation of Liver VX2 Tumors in Rabbits

VX2 tumor cells (~1×10^6^) were implanted into the thigh muscles of donor rabbits. Approximately two weeks later, the donor rabbits with VX2 tumors in thigh muscles were euthanized with intravenous injection of pentobarbital (100mg/kg body weight). The tumors were harvested and viable tumor tissues were minced into 1–2 mm^3^ pieces under sterile conditions.

Using the disinfection approach, a 3–4 cm incision was made below the sub-xiphoid process to expose the left lobe of the liver. Then, a 1 mm^3^ tumor fragment, collected from the donor VX2 tumor, was directly implanted into the subcapsular parenchyma of the liver lobe. This was followed by compression of the tumor injection site with a gelatin sponge (Pharmacia & Upjohn Co; Kalamazoo, MI) for 5 min and closure of the incision with sutures.

### Treatment of Liver VX2 Tumors

At day 14 after the tumor implantation, the average size of the tumors just before RFH-enhanced treatment was 1.0 ± 0.47cm^3^. Twenty-four rabbits with tumors were randomly assigned in four groups (n=6/group), receiving different treatments: (1) combination therapy of peri-tumoral infusion of DOX (4 mg/Kg) plus RFH ablation; (2) DOX alone; (3) RFH alone; and (4) PBS as the control group.

RFH was performed by attaching a custom-made 0.022-inch radiofrequency heating wire onto the bottom of the chamber and connecting it to a radiofrequency generator (RF field at the power of 400 KHz, 42°C). A sterilized 1.1 mm fiberoptic temperature probe was placed in the bottom of each chamber and connected to a thermometer (PhotonControl, Burnaby, British Columbia, Canada) for real-time monitoring of the temperatures during the RFH.

**Figure 2:**
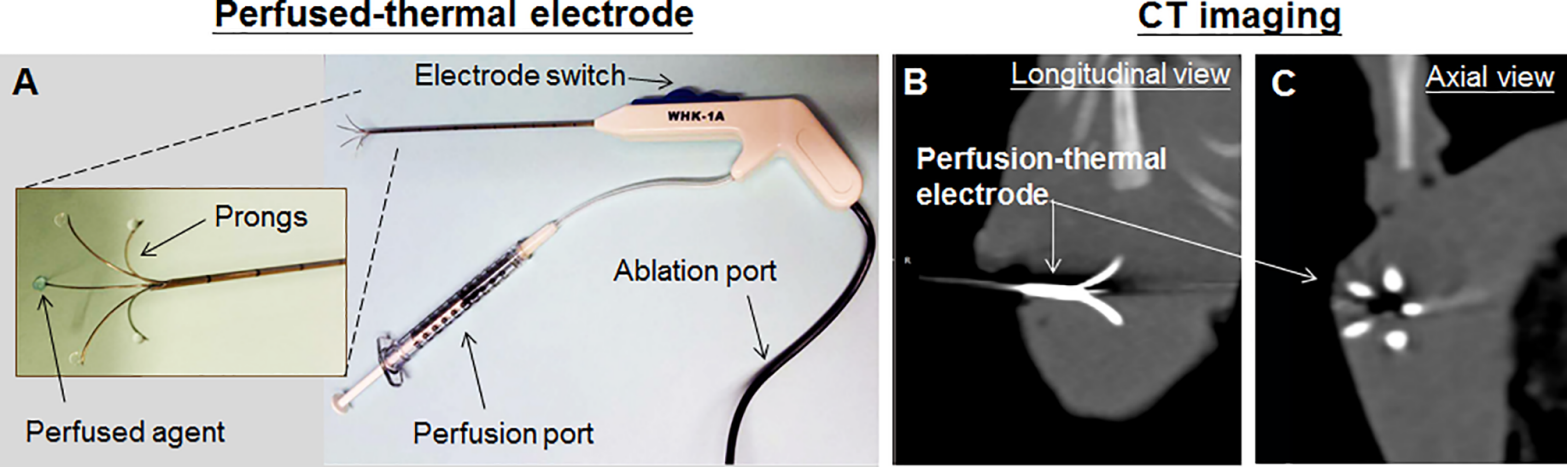
**(A)** The perfusion-thermal RF electrode. There is a tunnel within each of electrode prongs, which enables to perfuse agents into the ablated tumor periphery *via* the perfusion port. **(B, C)** The deploy size of the electrode prongs can be controlled *via* the electrode switch and precisely monitored under CT.

DOX was infused into the peritumoral region through a multi-functional perfusion-thermal RFA electrode system which was capable of delivering RF-induced thermal energy and chemotherapeutic drugs in the peritumoral areas of rabbit liver VX2 tumors (**Figure 2**). The sizes of the ablation and/or hyperthermic zones created by the RFA probe was the same among tumors, as we covered the entire tumor margin and injected the DOX into the margin, which were precisely controlled by the real-time ultrasounding.

### Western Blotting Assay for Quantifying HSP70 in Tumor Tissues

After homogenization of liver tissue, total protein was extracted using protein extraction buffer, subjected to Western blotting analysis according to the manufacturer protocol (TermoFisher Scientific). The level of HSP70 expression was quantified by measuring the density of the immunoreactive bands.

### Immunohistochemistry

All tumor specimens were fixed in 10% neutral buffered formalin, embedded in paraffin and sectioned with a microtome, for immunohistochemical analysis. Immunoreactive proteins were visualized using a chemiluminescence protocol (DoGEN ECL, Daeil Lab Service; Seoul, South Korea). Slides were heated in citrate buffer (0.01 M, pH 6.0) for 16 min in a microwave oven and endogenous peroxidase was blocked with methanol containing 3% hydrogen peroxide for 10 min. For immunohistochemical detection of HSP70, the specimens were incubated overnight at 4°C with mouse anti-HSP70 (ab2787, Abcam; Cambridge, MA) monoclonal antibodies and rabbit Anti-Ki67 polyclonal antibody (ab15580, Abcam), respectively. The sections were then incubated with an anti-mouse secondary antibody (Dako) for 30 min at room temperature and binding reactions were visualized by DAB (3-30-diaminobenzidine tetrahydrochloride) substrate. The nucleus was lightly counterstained with hematoxylin. An Apoptosis Detection Kit (R&D Systems; Minneapolis, MN) was used to evaluate apoptosis and terminal deoxynucleotidyl transferase dUTP nick end labeling (TUNEL) assay was carried out according to the manufacturer’s protocol. The relative signal intensity was quantified by densitometry with Image-Pro Plus 6.0 software (Media Cybernetics; Silver Spring, MD) and the integrated optical density (IOD) was used to present the relative levels of Hsp70, TUNEL, and Ki67. The IOD of the fluorescence or number of positive cells per 100 μm2 was evaluated throughout the area.

### Statistical Analysis

All data were expressed as mean ± standard deviation and further statistical analysis was carried out by using Prism 5.0 (GraphPad Software; La Jolla, CA). Differences between treatment groups were compared with Mann-Whitney test and analysis of variance (ANOVA) associated with Tukey’s multiple comparisons test. P<0.05 was considered statistically significant.

## Results

### Hyperthermia-Enhanced DOX Cell Killing Effect

**Figure 3:**
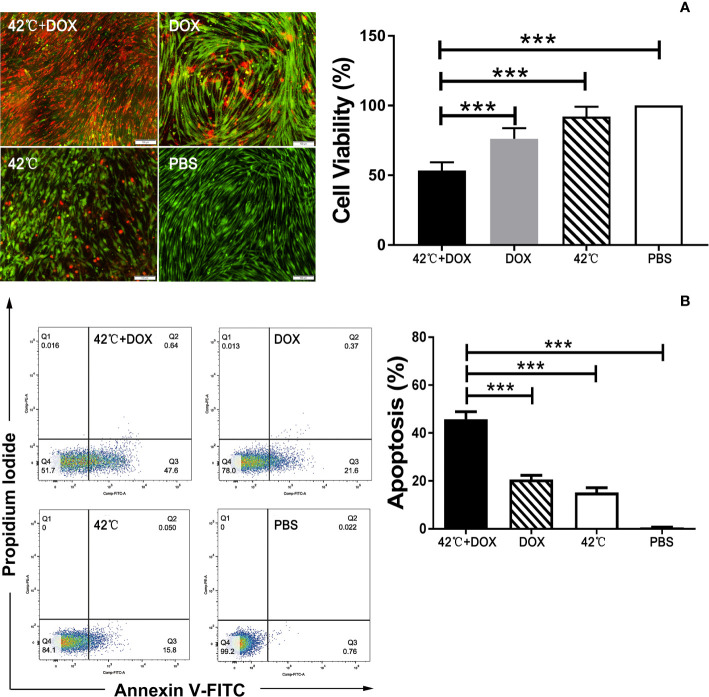
**(A)** Fluorescence microscopy of VX2 tumor cells with Live/Dead staining showing more dead cells (red) and less viable cells (green) in the combination treatment of DOX plus 42°C hyperthermia compared to other control groups of DOX alone, 42°C hyperthermia alone, and PBS. MTS assay demonstrating the lowest cell viability in the combination therapy of DOX plus 42°C hyperthermia compared to other treatments (***p<0.001). **(B)** Flow cytometry showing the combination treatment with DOX plus 42°C hyperthermia induced early apoptosis of VX2 cells compared with other treatments (***p<0.001).

Live/Dead staining of VX2 cells showed more cell death (red ethidium bromide staining) and less cell viability (green calcein staining) in the group treated with the combination therapy of DOX plus 42°C hyperthermia. MTS assay confirmed the lowest cell viability with combination therapy compared to the other treatments of DOX alone, 42°C hyperthermia alone, and PBS (53.47 ± 2.39% vs 76.23 ± 3.12% vs 92.03 ± 2.93% vs 100%, respectively, p<0.001) (**Figure 3A**).

Flowcytometry of VX2 cells stained with annexin V/propidium iodide (PI) showed more apoptotic cells in the combination therapy of DOX plus 42°C hyperthermia compared to DOX alone, 42°C hyperthermia alone, and PBS (45.80 ± 1.27% vs 20.66 ± 0.71%, vs 15.16 ± 0.81% and 0.62 ± 0.06%, respectively, p<0.01 (**Figure 3B**).

### Hyperthermia-Enhanced Intracellular Uptake of Doxorubicin

**Figure 4:**
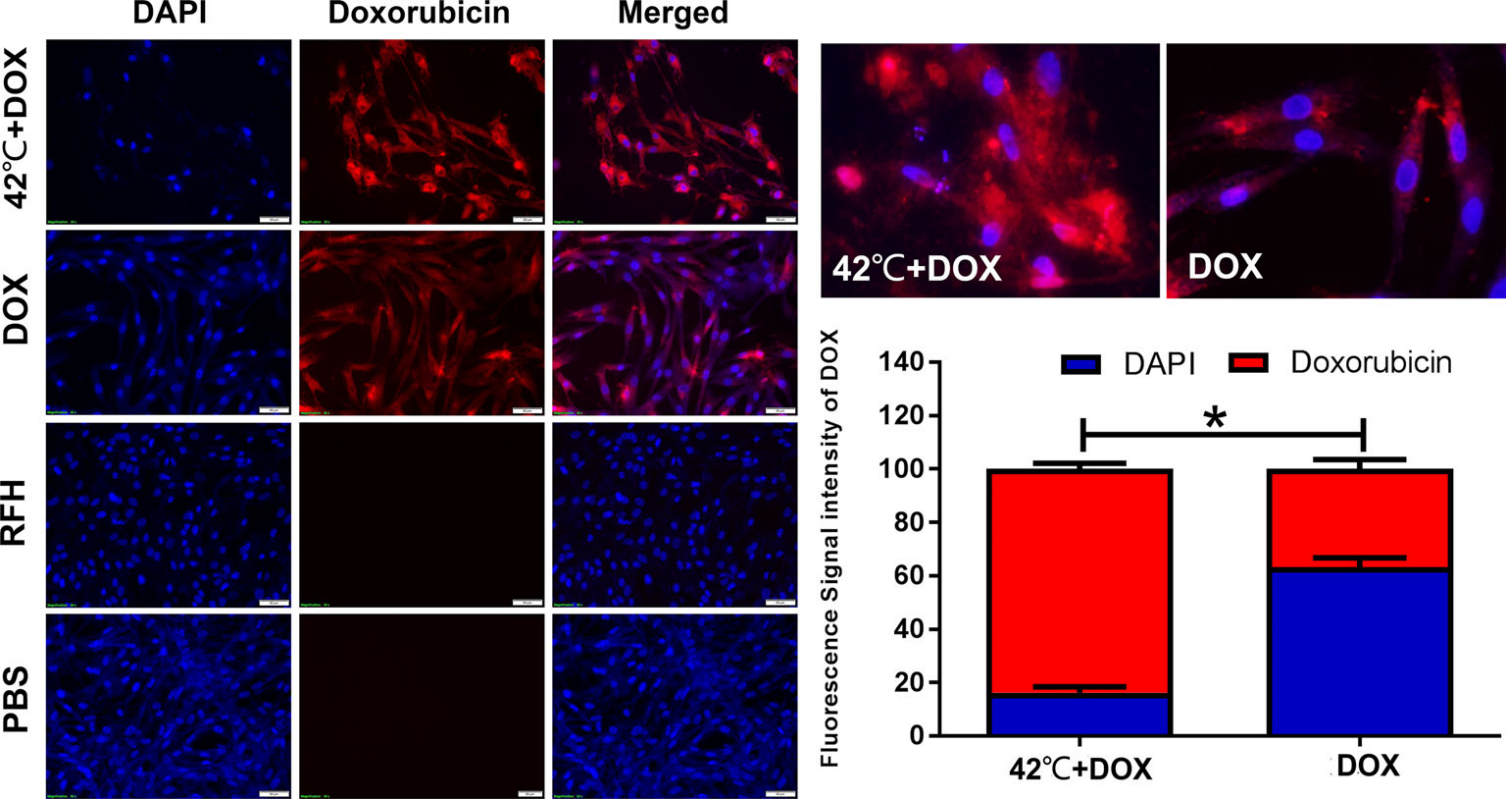
Confocal microscopy showing increased red fluorescence intensity of doxorubicin (red) in cells treated with the combination therapy of DOX plus 42°C hyperthermia, compared to other treatments (Right) (*p<0.05).

Confocal microscopy showed higher red fluorescence signals emitted by doxorubicin in VX2 tumor cells that were treated with DOX in combination with hyperthermia and that hyperthermia increased the uptake of DOX by cells (83.79% ± 2.17 vs. 36.85% ± 3.49, p<0.05) (**Figure 4**).

### RFH -Enhanced Distribution of Doxorubicin in the VX2 Tumor Margin

**Figure 5:**
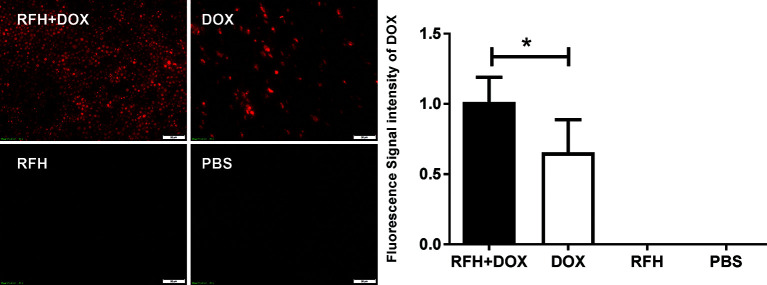
Fluorescence microscopy of VX2 tumor tissues showing the fluorescence of DOX in the peri-tumoral region was increased with the simultaneous delivery of RF DOX plus 42°C hyperthermia compared with the delivery of doxorubicin alone (*p<0.05).

Fluorescence intensity increased in tumors treated with delivery of 42°C hyperthermia and DOX compared to DOX alone, resulting in the integrated optical density (IOD)/Unit Area at 1.01 ± 0.07 vs 0.65 ± 0.10, p<0.05) (**Figure 5**).

### RFH-Enhanced Apoptosis of VX2 Liver Tumors

**Figure 6:**
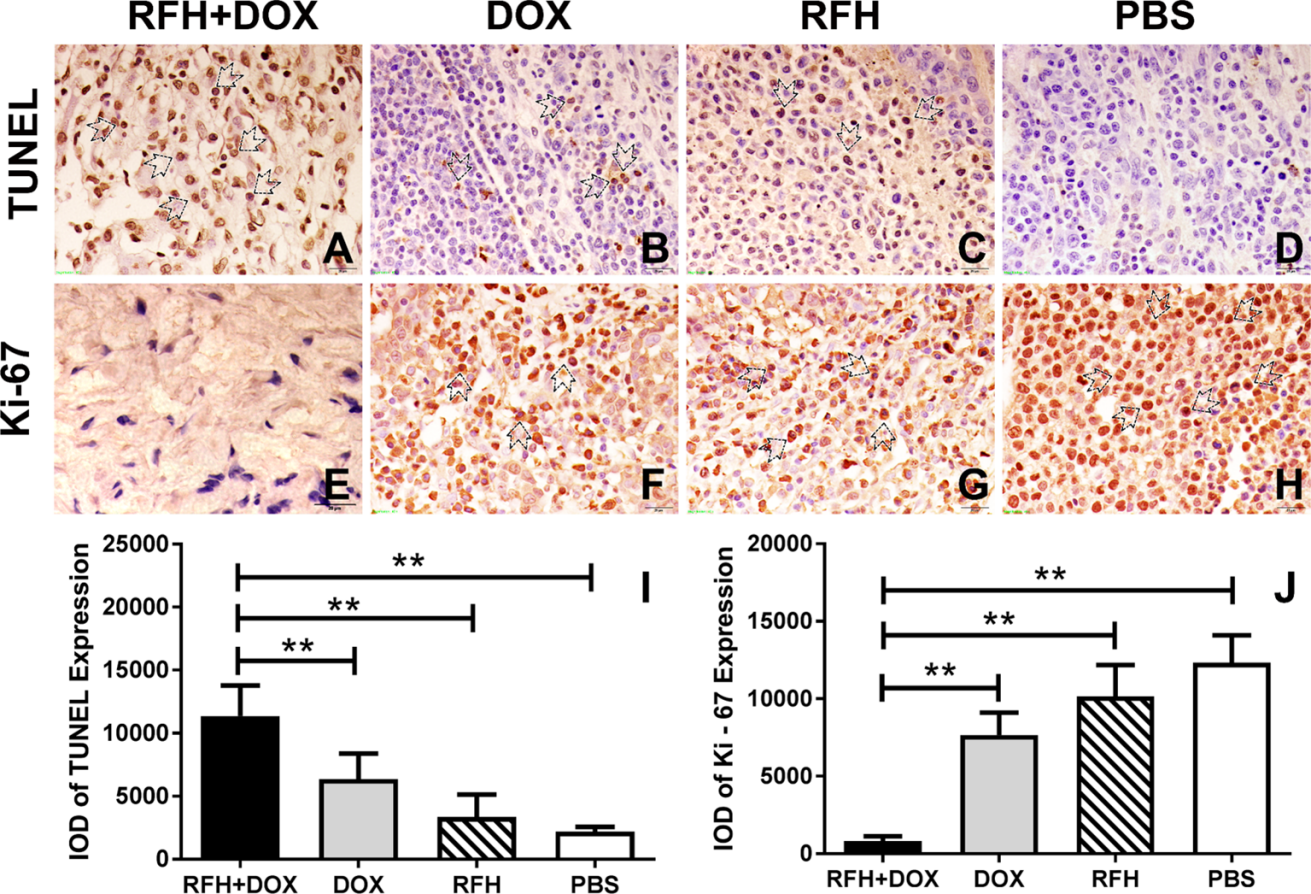
Immunohistochemistry showing that the treatment with DOX plus RFH induced early apoptosis and inhibited the proliferation of VX2 cells compared with other treatments (**p<0.01).

TUNEL staining showed that the number of apoptotic cells was the highest in combination therapy group compared to DOX alone, RFH alone, and PBS (11,356.00 ± 986.60 vs 6,369.21 ± 826.14 vs 3,371.20 ± 720.50 vs 2,200.01 ± 154.30, respectively, p <0.01). Correspondingly, immunohistochemistry staining of Ki-67, reflecting the proliferation of cells, showed that the proliferation in the combination therapy group was the lowest among the four groups (800.00 ± 129.5 vs 7,666.00 ± 586.50 vs 10,156.51 ± 823.30 vs 12,319.13 ± 721.20, respectively, p<0.01) (**Figure 6**).

### Hyperthermia/RFH-Enhanced HSP70 Expression in VX2 Cells and VX2 Liver Tumors

**Figure 7:**
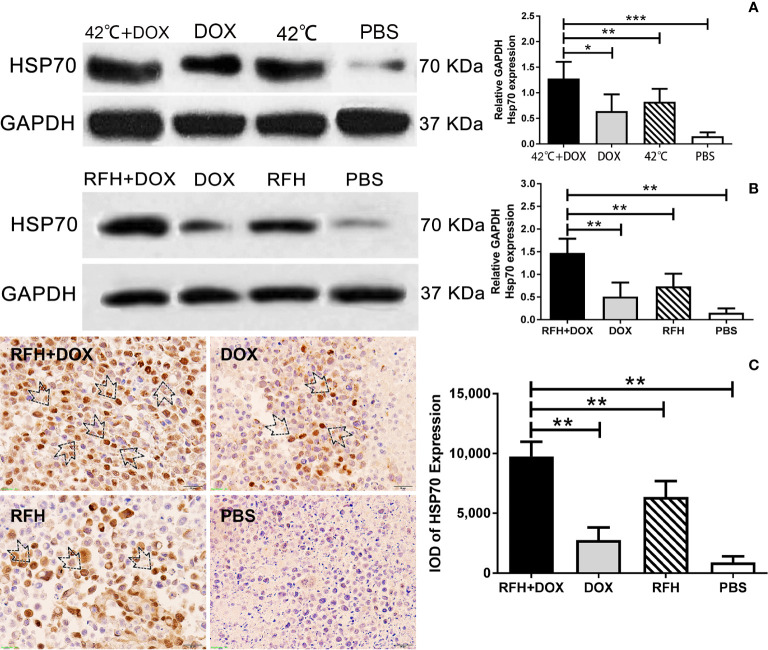
**(A)** Western blot analysis showing that treatment with DOX plus 42°C hyperthermia enhanced the expression of HSP70 in VX2 tumor cells compared with the other treatments (***p<0.001; **p<0.01; *p<0.05). **(B)** Western blot confirmed that treatment with DOX plus 42°C hyperthermia induced HSP70 expression in VX2 tumor tissues compared with control treatments. (**p<0.01). **(C)** Immunohistochemistry showing that the treatment with DOX plus RFH increased HSP70 expression in VX2 tumor tissues (**p<0.01).

HSP70 expression (relative to GADPH) was increased when cells were treated with the combination of DOX plus RFH compared to DOX alone, RFH alone, and PBS (1.28 ± 0.13 vs 0.64 ± 0.13 vs 0.83 ± 0.10 vs 0.15 ± 0.03, respectively, p<0.05) (**Figure 7A**). Western blot further confirmed the increase of HSP70 expression (relative to GADPH) in the tumors with DOX plus RFH treatment compared to other treatments of DOX alone, RFH alone, and PBS (1.47 ± 0.13 vs 0.51 ± 0.13 vs 0.74 ± 0.11 vs 0.16 ± 0.04, respectively, p <0.01) (**Figure 7B**).

Immunocytochemical staining was performed to determine the expression level of HSP70 in tumors. An increase of HSP70 staining was found in tumors treated with simultaneous DOX plus RFH compared to other groups (9,756.00 ± 274.25 vs 2,758.03 ± 237.70 vs 6,372.42 ± 294.73 vs 895.70 ± 113.20, respectively, p <0.01) (**Figure 7C**).

## Discussion

RFA is a minimally invasive treatment for liver tumors. In the ablation region, coagulation necrosis may be seen (6). The electrical insulation effect of tissue, in addition to heat-sink effects caused by blood flow adjacent to tumors, may; however, create thermal energy decay. Consequently, tumor cells in the margin of large tumors may not be completely eradicated (7).

Previous studies have shown that RFH may enhance the direct intra-tumoral chemotherapy effects on human cancers (8, 9). Though HSP70 has been shown to be a potent activator of innate immunity, the immunological consequences mediated by their expression in RFH-treated tumors have not been explored.

This study used a multifunctional RF electrode to treat liver cancers. Moreover, this study used sublethal thermal energy in the margin of tumors to boost the chemotherapeutic effects of DOX (10). 42°C hyperthermia enhanced DOX uptake two fold by VX2 cells. At the *in vivo* situation, one of the primary reasons for increased uptake in tumors is related to the increased diffusion of DOX into tumor due to leaky of vasculatures by mild hyperthermia.

The increased cytotoxicity of DOX, when administered with heat, may be attributed to modulating the tumor physiology and microenvironment, due to the increase of heat-induced membrane permeability and concomitant cellular uptake, which thus increases drug accumulation within the tumor cells (11, 12).

This study also found RFH/42°C hyperthermia, at 42–45°C, in combination with local chemotherapy, increased HSP70 expression in VX2 cells and VX2 tumor tissue. Adkins et al. (13) evaluated the effects of heat-shock treatments and found that the mechanisms of immunogenicity were largely attributed to HSPs when tumor cells were undergoing heat shock response. HSP70 immunomodulatory resulted in a T cell-mediated immune responses and increased CD8+ T cells and induced effector and memory T cells. The therapeutic effect was strengthened by the increased infiltration of specific CD4+ and CD8+ T lymphocytes and activation of cytotoxic NK cells (14). HSP70 promoted the release of pro-apoptotic factors and facilitated mitochondrial membrane permeability. The range of immunomodulatory activities of HSP70 makes it an ideal target for anticancer immunotherapy (15).

Although this study provided an insight into the underlying mechanism of RFH-enhanced chemotherapy in liver tumors, the present study has several limitations. The authors failed to follow the animals to the time point when the treated tumors completely disappeared as longer follow-up would result in the tumor size exceeding ten percent of the body weight which would not be allowed by the Institutional Animal Care and Use Committee. Unfortunately, the molecular mechanisms for hyperthermia-enhanced cell uptake of DOX are still not fully understood. And the potential immune response activation is a hypothesis based on previously reported results involving HSP70 activation, this study failed to explore the immunogenicity induced by HSP70 in tumors due to the lack of specific antibodies for rabbit T lymphocytes. Further clinical studies may be necessary to gain insight into the potential immunological mechanism of hyperthermia-enhanced local chemotherapy.

In conclusions, this study showed that hyperthermia at the margin of a RF-ablation enhanced peri-tumoral chemotherapy in rabbit liver VX2 tumors. RFH/42°C hyperthermia enhanced the chemotherapeutic effect of DOX in VX2 tumors by promoting uptake of DOX in cells and the expression of HSP70.

## Data Availability Statement

The original contributions presented in the study are included in the article/supplementary materials. Further inquiries can be directed to the corresponding authors.

## Ethics Statement

The animal study was reviewed and approved by Institutional Animal Care and Use Committee of the University of Washington (IACUC).

## Author Contributions

Conceptualization, KQ and XY. Validation, KQ, MC, and FZ. Methodology, formal analysis, and investigation, KQ. Resources, FZ and XY. Data curation KQ and XY. Writing—original draft preparation, KQ and FZ. Writing—review and editing, FZ, JC, HJ, CZ, and XY. Visualization, KQ and FZ. Supervision, CZ and XY. Project administration, XY. Funding acquisition, KQ and XY. All authors have read and agreed to the published version of the manuscript. All authors contributed to the article and approved the submitted version.

## Funding

This research was funded by the NIH RO1EBO12467 and NIH R01EB028095 grants, and a grant from the National Natural Sciences Foundation of China, grant number 81701800.

## Conflict of Interest

The authors declare that the research was conducted in the absence of any commercial or financial relationships that could be construed as a potential conflict of interest.
